# Nitric Oxide Regulates Seedling Growth and Mitochondrial Responses in Aged Oat Seeds

**DOI:** 10.3390/ijms19041052

**Published:** 2018-04-02

**Authors:** Chunli Mao, Yanqiao Zhu, Hang Cheng, Huifang Yan, Liyuan Zhao, Jia Tang, Xiqing Ma, Peisheng Mao

**Affiliations:** 1 College of Animal Science and Technology, China Agricultural University, Beijing 100193, China; maochunlim@163.com (C.M.); yanq_zhu@cau.edu.cn (Y.Z.); chenghang0522@126.com (H.C.); yanhui_fang@126.com (H.Y.);yuanlizhao1994@126.com (L.Z.); candytangjia@cau.edu.cn (J.T.); ma2016@cau.edu.cn (X.M.); 2 Key Laboratory of Pratacultural Science, Beijing Municipality, China Agricultural University, No. 2 Yuanmingyuan West Road, Haidian District, Beijing 100193, China

**Keywords:** nitric oxide, ROS, mitochondria, proteins, alternative pathway, antioxidant enzymes

## Abstract

Mitochondria are the source of reactive oxygen species (ROS) in plant cells and play a central role in the mitochondrial electron transport chain (ETC) and tricarboxylic acid cycle (TCA) cycles; however, ROS production and regulation for seed germination, seedling growth, as well as mitochondrial responses to abiotic stress, are not clear. This study was conducted to obtain basic information on seed germination, embryo mitochondrial antioxidant responses, and protein profile changes in artificial aging in oat seeds (*Avena sativa* L.) exposed to exogenous nitric oxide (NO) treatment. The results showed that the accumulation of H_2_O_2_ in mitochondria increased significantly in aged seeds. Artificial aging can lead to a loss of seed vigor, which was shown by a decline in seed germination and the extension of mean germination time (MGT). Seedling growth was also inhibited. Some enzymes, including catalase (CAT), glutathione reductase (GR), dehydroascorbate reductase (DHAR), and monodehydroascorbate reductase (MDHAR), maintained a lower level in the ascorbate-glutathione (AsA-GSH) scavenging system. Proteomic analysis revealed that the expression of some proteins related to the TCA cycle were down-regulated and several enzymes related to mitochondrial ETC were up-regulated. With the application of 0.05 mM NO in aged oat seeds, a protective effect was observed, demonstrated by an improvement in seed vigor and increased H_2_O_2_ scavenging ability in mitochondria. There were also higher activities of CAT, GR, MDHAR, and DHAR in the AsA-GSH scavenging system, enhanced TCA cycle-related enzymes (malate dehydrogenase, succinate-CoA ligase, fumarate hydratase), and activated alternative pathways, as the cytochrome pathway was inhibited. Therefore, our results indicated that seedling growth and seed germinability could retain a certain level in aged oat seeds, predominantly depending on the lower NO regulation of the TCA cycle and AsA-GSH. Thus, it could be concluded that the application of 0.05 mM NO in aged oat seeds improved seed vigor by enhancing the mitochondrial TCA cycle and activating alternative pathways for improvement.

## 1. Introduction

High-quality seeds are extremely important to agricultural production, productivity, and germplasm conservation. However, seed deterioration occurs during storage, even under optimal storage conditions, which causes the loss of seed vigor. Various biochemical and metabolic alterations take place during seed aging, including electrolyte leakage, the loss of cell membrane integrity, DNA alteration, and damage of mitochondrial structure and function [1,2,3]. Although the mechanisms of seed aging are still being researched, reactive oxygen species (ROS) are considered the main factor contributing to seed aging and leading to the damage of lipids, DNA, and proteins [4,5]. ROS, including hydrogen peroxide (H_2_O_2_), hydroxyl radical (·OH), and superoxide radical (O_2_^−^), accumulate in the aged seeds of sunflowers (*Helianthus annuus* L.) [6], oats [2], and elm (*Ulmus pumila* L.) [7]. Therefore, it is necessary to explore the aging mechanisms of the detrimental role of ROS in deteriorated seeds.

Mitochondrion can provide energy for cell metabolism and transport by respiration and is the main site for the generation and scavenging of ROS [8,9,10]. Plant mitochondria have two different pathways for electron transport at the ubiquinone pool, the cyanide-sensitive cytochrome pathway and the cyanide-resistant alternative pathway. The cytochrome pathway, consisting of complex I (NADH dehydrogenase), complex II (succinate dehydrogenase), complex III, and finally complex IV (cytochrome oxidase), catalyze the four-electron reduction of O_2_ to H_2_O [11]. However, complex I and complex III are considered as the main source of ROS [8,12]. There are two terminal oxidases in the plant mitochondrial electron transport chain (ETC). In addition to the cytochrome pathway, alternative oxidase (AOX) can be used as terminal oxidase to reduce O_2_ to H_2_O in the alternative respiratory pathway descried in plant mitochondria and could produce a branch in the ETC. Then, electrons in ubiquinone are divided between the cytochrome pathway (complex III and complex IV) and AOX [11,13]. It has been reported that severe drought stress induces the accumulation of ROS in wheat (*Triticum aestivum* L.) seedlings; however, alternative pathways could improve drought-resistance by removing ROS [14]. Over-expression of the *AOX* gene reduces the level of ROS in *Arabidopsis* under chilling stress, while suppressing *AOX* induces higher levels of ROS [15]. Thus, studies have shown that AOX could play important role in balancing ROS during plant oxidative stress. However, the relationship between the alternative pathway and ROS accumulation in the mitochondria of aged seeds has not yet been thoroughly studied.

Mitochondria are important sites for the scavenging of ROS, consisting of the enzymatic antioxidant systems and non-enzymatic antioxidant systems, such as superoxide dismutase (SOD), catalase (CAT), and ascorbate-glutathione (AsA-GSH) cycles [16,17,18]. It has been shown that the activities of antioxidant enzymes decrease as a result of seed aging [2,6]. However, there is no further information on the role of different antioxidant enzymes on ROS scavenging in mitochondria.

Nitric oxide (NO) is a gaseous signaling transduction molecule and plays an important role in responding to diverse stressors in plants. It has been suggested that NO is a regulator of germination as well as H_2_O_2_ [5,19]. Some research has provided evidence that many of the crucial physiological processes of plants are related with NO, including germination, respiration, stress response, and regulating ROS balance. Exogenous NO could significantly enhance the germination rate of wheat seeds and decreased the content of H_2_O_2_ and O_2_^−^ in the mitochondria under salt stress [20]. NO treatment has been shown to improve the activities of CAT, SOD, and APX in cucumber (*Cucumis sativus* L.) under salt stress [21] and wheat seed under copper stress [22]. Moreover, NO could inhibit the cytochrome pathway, while inducing the alternative pathway [23]. Royo et al. [24] showed that NO is essential for the induction of the AOX pathway under phosphate-limiting conditions in *Arabidopsis*. However, there is little known about the role of NO in regulating mitochondrial ROS and antioxidant mechanisms in aged seeds.

Recently, proteomics has become an important method to study the mechanisms of aged seeds and detect changes in various cellular processes. Li et al. [7] identified 48 mitochondrial proteins that changed in abundance on aged elm seeds and found that the alteration of voltage-dependent anion channels (VDAC), tricarboxylic acid cycle (TCA), and mitochondrial ETC were related to seed aging. Yin et al. [25] revealed the mitochondrial metabolite of aged rice (*Oryza sativa* L.) seeds under the critical node and found that induction of the alternative pathway led to a decrease in cytochrome c and the accumulation of ROS. However, the way in which events are regulated in the mitochondria of aged seeds should be further studied.

Oat, a low-carbon and eco-friendly crop, can be planted in regions experiencing a variety of environmental stresses, including infertility, salinity, drought, and cold. This study was designed to determine changes in mitochondria as a result of exogenously applied NO, including seedling growth, ROS accumulation, antioxidant enzyme improvement in the AsA-GSH cycle, and proteomics information in the embryo of oat seeds after aging, and to understand the response of mitochondria to seed deterioration.

## 2. Results

### 2.1. Changes in Seed Germination and Seedling Growth in Aged Oat Seeds under Nitric Oxide Treatments

**Figure 1:**
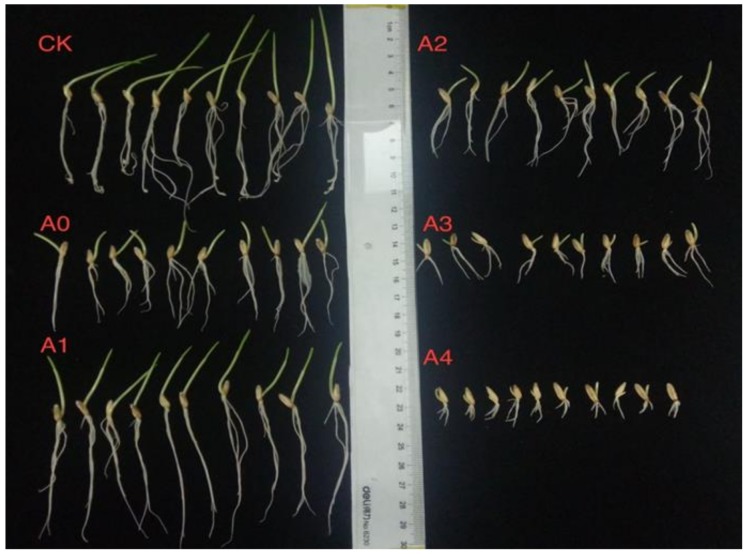
Patterns of seedling length in aged oat seeds with different NO treatments.

The germination percentage and seedling length of aged oat seeds (A0) was significantly (*p* < 0.05) lower than non-aged seeds (CK), and improved significantly (*p* < 0.05) after NO treatment (A1) in all areas except for seed germination (Table 1). On the other hand, there were severe inhibiting roles of higher NO contents (A3 and A4, Figure 1) on root and shoot length. Mean germination time (MGT) of aged oat seeds (A0) was significantly higher (*p* < 0.05) than CK and could also be significantly (*p* < 0.05) reduced with NO treatment (A1). However, there were no effect of higher NO content (A2 and A3) on MGT (Table 1).

### 2.2. Changes in Mitochondrial H_2_O_2_ Content, Malate Dehydrogenase (NAD-MDH), and O_2_^−^ Content in Aged Oat Seeds under NO Treatments

**Figure 2:**
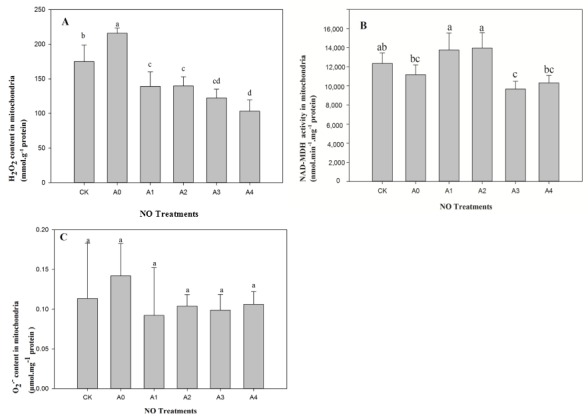
Changes in mitochondrial H_2_O_2_ content and NAD-MDH activity in aged oat seeds with different NO treatments. (**A**) H_2_O_2_ content; (**B**) NAD-MDH activities; (**C**) O_2_^−^ content. Data are means ± SD from three replications for each treatment. Different letters indicate significant differences among NO treatments (*p* < 0.05).

The content of mitochondrial H_2_O_2_ in aged seeds (A0) was significantly higher (*p* < 0.05) compared to CK, and NO application significantly (*p* < 0.05) decreased the content of H_2_O_2_ in aged seeds (Figure 2A). However, there were no significant differences (*p* > 0.05) in H_2_O_2_ content among the treatments of A1, A2, and A3, and H_2_O_2_ content reached the lowest point at A4.

There was no significant difference (*p* > 0.05) in terms of the activity of mitochondrial NAD-MDH between A0 and CK, but activities of NAD-MDH were significantly improved (*p* < 0.05) with lower level exogenous NO treatments (A1 and A2), compared to A0 (Figure 2B). Furthermore, activity decreased significantly (*p* < 0.05) as NO content increased from A1 to A4 and reached the lowest level at A3. There was no significant difference (*p* > 0.05) between A3 and A4, which were similar to A0 (Figure 2B).

The mitochondrial O_2_^−^ content in aged seeds (A0) increased compared to CK, and NO application decreased the content of O_2_^−^ in aged seeds (Figure 2C). However, there were no significant differences (*p* > 0.05) of O_2_^−^ content among the treatments of CK, A0, A1, A2, A3, and A4.

### 2.3. Changes in Mitochondrial Antioxidant Enzymes in Aged Oat Seeds under Nitric Oxide Treatments

**Figure 3:**
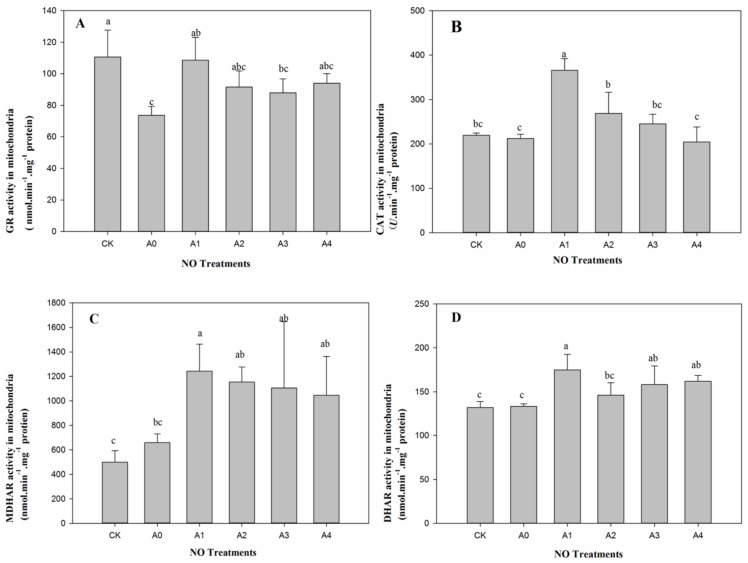
Changes in mitochondrial antioxidant enzyme activities in aged oat seeds with different NO treatments. (**A**) Glutathione reductase (GR) activities; (**B**) Catalase (CAT) activities; (**C**) Monodehydroascorbate reductase (MDHAR) activities; (**D**) Dehydroascorbate reductase (DHAR) activities. Data are means ± SD from three replications for each treatment Different letters indicate significant differences among NO treatments (*p* < 0.05).

There was a significant difference (*p* < 0.05) in the activity of mitochondrial glutathione reductase (GR) between A0 and CK, but no differences were found in terms of CAT, monodehydroascorbate reductase (MDHAR), and dehydroascorbate reductase (DHAR) (Figure 3). Furthermore, activities of GR, CAT, MDHAR, and DHAR were all significantly improved (*p* < 0.05) with lower level of exogenous NO treatment (A1), compared to A0. However, enzyme activities displayed different tendencies as NO application increased from A1 to A4. The activities of GR, MDHAR, and DHAR presented no significant increase among NO treatments from A1 to A4 (Figure 3A,C,D). For CAT, activity decreased significantly (*p* < 0.05) with increasing exogenous NO content and reached the lowest level at A4 treatment, which showed no significant difference to CK or A0 (Figure 3B).

### 2.4. Changes in Mitochondrial Complex IV in Aged Oat Seeds under Nitric Oxide Treatments

**Figure 4:**
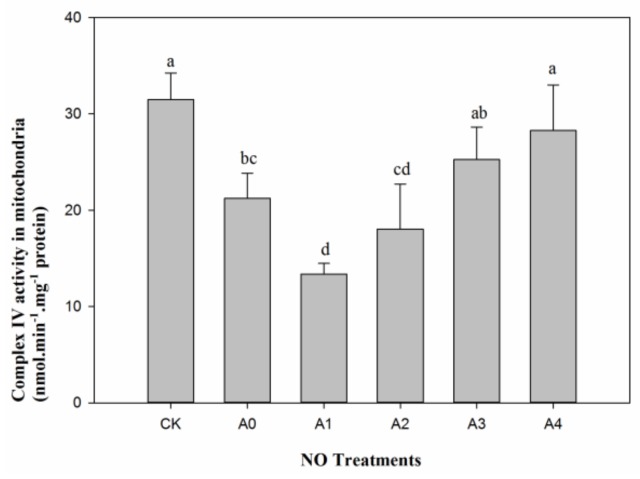
Changes in mitochondrial complex IV activity in aged oat seeds with different NO treatments. Data are means ± SD from three replications for each treatment. Different letters indicate significant differences among NO treatments (*p* < 0.05).

The activity of mitochondrial complex IV in aged oat seeds (A0) decreased significantly (*p* < 0.05) compared to un-aged seeds (CK). There were different effects for different NO contents on the activity of mitochondrial complex IV in aged seeds. The activity of mitochondrial complex IV in the aged seeds significant decreased (*p* < 0.05) at the lower NO treatment level (A1), then activity began to increase with NO treatments from A2 to A4. The level of activity of mitochondrial complex IV under A3 and A4 treatments attained the highest level and returned to a similar level to CK (Figure 4).

### 2.5. Changes in Mitochondrial Proteins in Aged Oat Seeds under Nitric Oxide Treatments

According to the results of H_2_O_2_ and antioxidant enzymes in aged oat seeds treated with NO, we found that A1 treatment was the most sensitive in terms of mitochondrial physiology. Therefore, seed samples from CK, A0, and A1 were selected for the proteomics analysis.

A total of 3874 proteins were found in the quantitative analysis of proteomics. Of the proteins screened with mitochondrion markers, 103 mitochondrial related proteins were identified. At the same time, the results of Gene Ontology (GO) annotation showed that 37 proteins were enriched in mitochondrial related pathways, and there were four overlapping proteins found between mitochondrial markers and GO annotation. Therefore, a total of 136 mitochondrial proteins could be identified. In order to screen the differentially expressed proteins depending on the fold changes, we divided the results of the proteomic analysis into three comparison groups, including A1/A0 (0.05 mM sodium nitroprusside (SNP) treatment compared to 0 mM SNP in aged seeds), A0/CK (aged seeds compared to unaged seeds), and A1/CK (0.05 mM sodium nitroprusside treatment in aged seeds compared to unaged seeds). 

**Figure 5:**
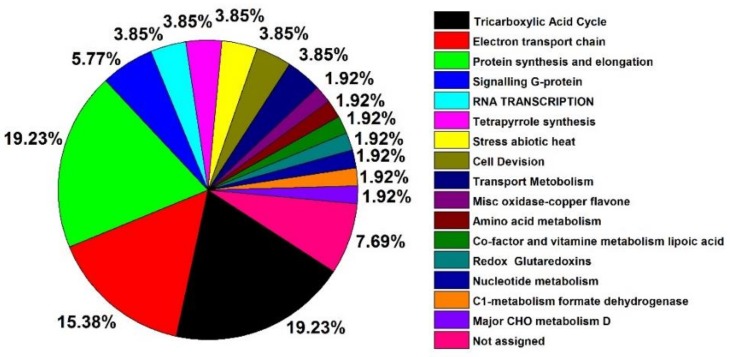
The functional category distribution of the 52 differently expressed mitochondrial proteins in aged oat seeds. Note: Functional classification was based on the MapMan.

In general, only 52 differentially expressed proteins were determined for their name and functions according to the MapMan (Table 2). For A1/A0, 11 differentially expressed proteins were identified, including eight up-regulated and three down-regulated proteins. There were 11 up-regulated proteins and 29 down-regulated proteins in A0/CK, and 11 up-regulated and 24 down-regulated proteins in A1/CK. The number of up-regulated proteins was very similar, and there were much more down-regulated proteins in the group of A0/CK and A1/CK. It could be found that the number of up-regulated proteins were much more than that of down-regulated proteins in aged seeds with NO treatment (A1/A0). However, there were much more down-regulated proteins than up-regulated proteins with aging treatments in groups of A0/CK and A1/CK. According to the MapMan analysis, these differentially expressed proteins were classified into 12 functional categories, including TCA cycle, mitochondrial ETC, protein synthesis and elongation, signaling, RNA transcription, heat stress, cell division, and transport metabolism. Among these proteins, large numbers of differentially expressed proteins belonged to the functional categories of the TCA cycle and protein synthesis and elongation, which, as a proportion of total proteins, attained the highest level of 19.23%. The mitochondrial ETC followed this, at 15.38% (Figure 5).

**Figure 6:**
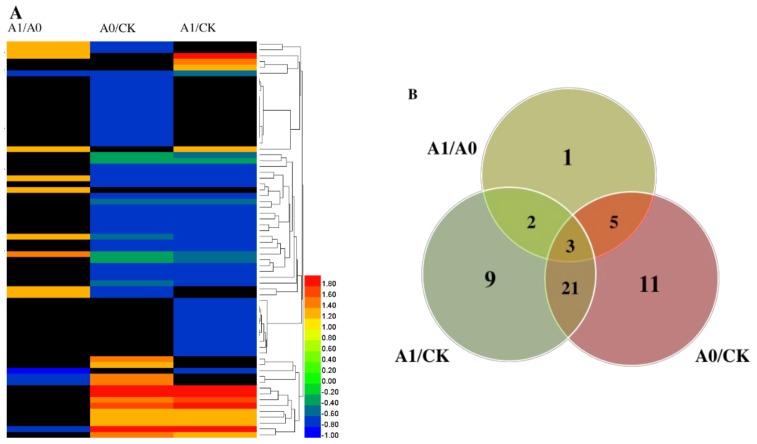
(**A**) Clustering analysis of differentially expressed proteins and (**B**) The number of differentially expressed proteins as a result of the exogenous application of NO across different groups (A1/A0, A0/CK, A1/CK) of aged oat seeds. Note: The color scale bar in the right of the hierarchical clustering analysis indicates the up-regulated (ratio > 0.00) and the down-regulated (ratio < 0.00) proteins. In the Venn diagram, the overlapping regions of cycles indicate proteins that were regulated in both or all treatments, whereas non-overlapping circles indicate proteins regulated in the only treatment.

Furthermore, from the overview of a hierarchical clustering analysis of the differentially expressed proteins in the three comparison groups, the differentially expressed proteins of A1/A0, A0/CK, and A1/CK showed different expression patterns (Figure 6A). Although there were three differentially expressed proteins identified in all three groups, two proteins existed in the groups of A1/A0 and A1/CK, the same 21 proteins in A1/CK and A0/CK, and the same five proteins were found in A1/A0 and A0/CK. Then, the remaining one, nine and 11 differentially expressed proteins only existed in the A1/A0, A1/CK, and A0/CK groups, respectively (Figure 6B). In particular, this occurred during the physiological processes, including the mitochondrial TCA cycle, ETC, and protein synthesis and elongation. Some proteins located in the TCA cycle were down-regulated in the aged seeds (A0/CK), such as the subunit of Succinate-CoA ligase (I1LYN0, K3ZV34, B4FRH5 and W5C4B7), fumarate hydratase (R4X771), malic enzyme (K3ZRI5), aldehyde dehydrogenase (K3YRJ0), and a subunit of Succinate-CoA dehydrogenase (M3AS20). However, the proteins of the subunit of Succinate-CoA ligase (I1LYN0 and K3ZV34) were only up-regulated in the aged seeds with NO treatment (A1/A0). Particularly for the Fumarate hydratase 1(Q10LR5), up-regulation appeared in seeds with NO treatment (A1/A0 and A1/CK). For the mitochondrial ETC proteins, the subunit of ATP synthase (W5BEP1 and A0A0K9R2N3) and external alternative NAD(P)H-ubiquinone oxidoreductase (Q9SKT7) were both up-regulated in the aged seeds (A0/CK) and with NO treatment (A1/CK); while, the subunit of ATP synthase (A0A200QHI3) was down-regulated in the aged seeds with NO application (A1/A0). For protein synthesis and elongation, aging treatment (A0/CK) could lead the proteins to become down-regulated, for example, the factor of elongation (A0A0D3H000, F2DG12, and F2EDF6), lon protease homolog (A0A0D3GV84 and W5C618); however, the factor of elongation (A0A0D3H000 and F2DG12) would be up-regulation with NO treatment (A1/A0). In the group of A1/CK, most proteins related to protein synthesis and elongation were down-regulated, except for the Chaperon in CPN60-like (Q8L7B5). Therefore, the proteins of the mitochondrial TCA cycle, mitochondrial ETC, and protein synthesis and elongation had different patterns across different groups (A1/A0, A0/CK, and A1/CK).

## 3. Discussion

Seed aging and deterioration during storage could induce the loss of seed vigor, and seed germination, especially MGT, is usually used to reflect the level of seed vigor. It has been reported that seed germination decreases in aged seeds, such as maize [26], oat [2], and elm [7]. In our study, seed germination percentage decreased significantly after 26 days of aging (Table 1). Exogenous NO promotes the germination of seeds under stress or no-stress conditions [27]. For example, NO has been shown to stimulate seed germination under severe salt stress in wheat [20], and NO pretreatment significantly improves seed germination under copper stress, also in wheat [21]. Accordingly, a lower level of NO treatment (i.e., group A1) could improve the seed germination percentage in aged oat seeds. However, the differences among A1, A2, and A3 were not significant, which meant that the improvement in seed germination was sensitive to lower contents of NO. Excessive content or no NO could depress seed germination. MGT, an important index of seed vigor, exhibited a strong vigor with a shorter time [28]. Meanwhile, MGT was dramatically shorter for the A1 compared to other treatments in the aged oat seeds, and the roots and seedlings were significantly elongated. The changes in MGT and seedling length illustrate that the aged oat seed reached a higher level of vigor with the application of 0.05 mM NO. Aging leads to a change in MGT during germination. It has been shown that the transcripts encoding the proteins are associated with protein synthesis and impart changes during germination [29]. Based on the influence of imbibing time on protein abundance, different imbibing time was selected for uniform size aged seeds and non-aged seeds to ensure that they were at the same physiological point.

Mitochondrion is important organelle for respiration and metabolism, including mitochondrial ETC, and the TCA cycle. The ETC, composed of a series of electron carriers on the inner membrane of the mitochondrion, is called a respiratory chain. Furthermore, the mitochondrial ETC is a main site for ROS production, especially complex I and complex III [8,12]. It was found that the content of H_2_O_2_ significantly increased in aged seeds, but O_2_^−^ content did not differ significantly, most likely because of the speed at which it was produced was not conductive to monitoring. Mitochondrial respiration might be disturbed and cause the over accumulation of ROS during seed storage. In this study, we found the mitochondrial NADH dehydrogenase [ubiquinone] flavoprotein 1 (A0A1J3KAQ0) in aged oat seeds (A0 and A1) was significantly lower than that in unaged seeds (CK). As a subunit of the mitochondrial membrane, respiratory chain NADH dehydrogenase (complex I), NADH dehydrogenase flavoprotein 1 (A0A1J3KAQ0), is believed to belong to the minimal assembly required for catalysis and complex I functions in the electron transfer from NADH to respiratory chain. The activity reduction of complex I limits the transmission of the mitochondrial respiratory chain. Jardim-Messeder et al. [30] illustrated that succinate dehydrogenase was an important site of ROS production in plant mitochondria, in addition to complex I and complex III, for enhancing H_2_O_2_ release, and could also be a limiting factor in plant growth through mitochondrial ROS generation. Li et al. [7] reported that the succinate dehydrogenase [ubiquinone] flavoprotein subunit 1 in mitochondrial ETC decreased in aged elm seeds. Furthermore, complex IV (Cytochrome c oxidase) was significantly lower in aged oat seeds compared to CK (Figure 5). Also, it was shown that NO could inhibit complex IV [31,32]. In our results, the activity of complex IV was significantly reduced only with a lower content of exogenous NO (A1), which reflected that the cytochrome respiratory chain was inhibited. ATP synthase (complex V) is responsible for ATP production in mitochondria. Different responses were found in terms of ATP synthase in oat seeds; the subunits of ATP synthase (W5BEP1 and A0A0K9R2N3) were up-regulated in the aged seeds (A0/CK), but no significant differences were observed for the NO treatment (A1/A0). The other two subunits (A0A200QHI3 and V4LNR7) were down-regulated with the NO treatment (A1/A0 and A1/CK). Plant mitochondria have two different pathways of electron transport: the cyanide-sensitive cytochrome pathway and the cyanide-resistant alternative pathway. With the administration of NO, the cytochrome pathway was inhibited and the alternative pathway might have changed. It has been reported that NO inhibited the cytochrome pathway, while the alternative pathway was not affected for soybean mitochondria [23].

Succinate dehydrogenase plays a central role in mitochondrion for linking the TCA cycle and ETC by catalyzing the succinate to fumarate. In our proteome analysis results, the subunit of Succinate-CoA ligase [ADP-forming] (I1LYN0 and K3ZV34) in aged seeds was significantly lower compared to CK. Succinate-CoA ligase can catalyze a reversible reaction, transforming succinyl-CoA and ADP or GDP to succinate and ATP or GTP [33]. Supplying 0.05 mM of NO (A1) significantly increased the Succinate-CoA ligase [ADP-forming] subunit (I1LYN0 and K3ZV34) in aged seeds. This protein is involved in the subpathway that synthesizes succinate from succinyl-CoA. Subsequently, succinate dehydrogenase catalyzes succinate to fumarate [30]. Then, the fumarase (fumarate hydratase; E.C. 4.2.1.20) catalyzes the reversible hydration of fumarate to malate, which is a part of TCA. The results showed that the protein abundance of fumarate hydratase (Q10LR5) in the A1 treatment was significantly higher than that of the aged seeds (A0). Meanwhile, NAD-MDH activity also increased dramatically with the supplying of 0.05 mM NO in aged seeds. MDH can catalyze the interconversion of malate and oxaloacetate, combined with the reduction or oxidation of the NAD pool [33]. NAD-MDH catalyzes NADH to reduce oxaloacetic acid and produce malate. The increase in fumarate hydratase and NAD-MDH suggested that there was an accumulation of malate. Malate can be oxidized by malic enzymes to yield pyruvate and CO_2_ through oxidative decarboxylation [34]. The mitochondrial pyruvate carrier (W5BQ98) protein was observably expressed in aged seeds with application of 0.05 mM NO (A1). It has been reported that pyruvate acts on AOX to stimulate its activity in mitochondria isolated from the roots of soybean seedlings [35]. Pyruvate could activate AOX in the mitochondria of soybean cotyledons [36] and tobacco leaf [37]. It has been shown that external administration of pyruvate to isolated mitochondria results in activation of AOX pathways in wild type and pyruvate kinase transgenic lines, and transgenic lines decrease pyruvate [38]. The results demonstrated that the AOX pathway was enhanced following the NO treatment.

In general, the cytochrome pathway and alternative pathway branches point to ubiquinone. Exogenous NO inhibits the cyanide-sensitive cytochrome pathway and induces the cyanide-resistant alternative pathway. This suggests complex III is a major site of ROS production in the mitochondrial electron transport chain [39]. Alternative pathways did not pass through complex III, which could decrease the production of ROS in aged seeds with the exogenous NO. On the other hand, there was evidence that AOX played a particularly important role in regulating the balance of ROS. The AOX pathway could decrease the accumulation of ROS [14,40], and there was a higher accumulation of ROS when AOX was suppressed [15]. NO could reduce the production of ROS by inhibiting the cytochrome pathway and inducing the alternative pathway, and the alternative pathway was activated by pyruvate. It has been showed that the presence of exogenous NO induces AOX [11,41]. This was consistent with our results, where NO activated AOX pathways and thus reduced the generation of ROS.

**Figure 7:**
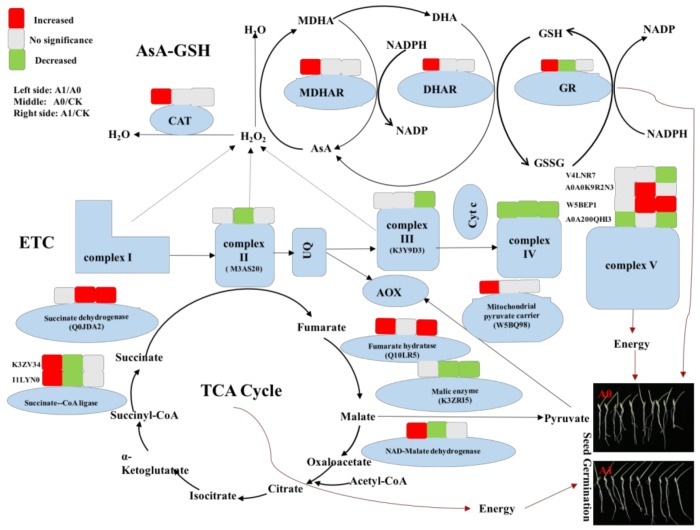
Schematic representation of protein abundance, ROS scavenging, and major biological pathways related to mitochondrial energy synthetic metabolism under NO treatment in aged oat seeds (different groups A1/A0, A0/CK, and A1/CK).

Based on the results obtained in this study, we proposed a possible schematic pathway which might be operating during artificial aging (Figure 7). During oat seed aging, the expression of some proteins related to the TCA cycle were down-regulated and several enzymes related to mitochondrial ETC were up-regulated. Additionally, H_2_O_2_ of ROS accumulated dramatically, and some enzymes, including CAT, GR, MDHAR, and DHAR maintained the lower level in the AsA-GSH scavenging system. Finally, seed germination and seedling growth were limited as oat seeds aged. This indicated that seed aging led to a decrease in the activities of GR, DHAR, and MDHAR, accompanied by a gradual reduction in the mitochondrial inner membrane [2]. Changes in mitochondrial structure are posited to be responsible for the decrease in antioxidant enzymes in aged seeds, however this is worth further study. In view of the results for NO treatment in aged seeds, some enzyme activities located in the TCA cycle (such as succinate-CoA ligase and fumarate hydratase) and AsA-GSH (such as CAT, GR, MDHAR, and DHAR) were enhanced and H_2_O_2_ content declined at lower level, before alternative pathways were activated as the cytochrome pathway was inhibited. Seedling growth of aged oat seeds could regain normality and seed germinability could be retained to a certain level. Taken together, our results clearly demonstrate that exogenous NO in aged oat seeds can enhance seed vigor by improving enzyme activities in the AsA-GSH and decreasing the accumulation of H_2_O_2_. Furthermore, the enhancement of the TCA cycle and activated alternative pathway are beneficial for seedling growth in aged oat seeds with the application of 0.05 mM NO.

## 4. Materials and Methods

### 4.1. Seed Materials

Oat seeds (variety: Haywire) were bought from Beijing Clover Seed & Turf Co. Ltd. (Beijing, China). The germination percentage of the seeds was 99% and original moisture content was 9.2%.

### 4.2. Determination of Seed Moisture Content

The seed moisture content was determined in accordance with ISTA (2015) [42]. Approximately 4.5 g of seeds were placed in a sample container and weighed, and then they were oven-dried at 130~133 °C for 2 h (two replicates). After cooling for 30 min in a desiccator, the seeds were weighed again and the moisture content was calculated.

### 4.3. Adjusting the Seed Moisture Content

The moisture content of seed samples was regulated to 10%. Approximately 160 g of seeds were placed in a desiccator with saturated potassium chloride solution and weighed continually. When the seed weight was required to reach the corresponding moisture content, they were immediately placed into an aluminum foil bag and sealed, then incubated at 4 °C for 1 day, at least.

### 4.4. Seed Aging Treatments

After regulation of seed moisture content, the seeds in the aluminum foil bags were aged in a constant temperature water bath at 45 °C, and then aged 26 days seeds were used for experimental samples. The aged seed germination percentage was 68%.

### 4.5. NO Treatment and Germination Test

Uniform sized seeds were used in all treatments. The aged seeds were treated with 0 (A0), 0.05 (A1), 0.1 (A2), 0.5 (A3), and 1.0 (A4) mM sodium nitroprusside (SNP) for 8 h in the germination incubator (GXZ-380B, Ningbo, China), and the non-aged seeds (CK) were treated with distilled water for 8 h in the germination incubator (GXZ-380B, Ningbo, China). Then, all treatments were rinsed three times with distilled water. The SNP acted as a donor of NO. The seed samples after NO treatment were used in the germination trials. Germination was assayed according to the rules of ISTA (2015) [42]. Four replicates of 100 seeds were germinated in Petri dishes on filter paper with distilled water. The test was conducted in the germination incubator (GXZ-380B, Ningbo, China) at 20 °C with 8 h light and 16 h dark for 10 days. On the 5th day, the length of the root and shoot were measured, and on the 10th day the number of normal seedlings was counted. When radicles emerged from the seed coat, the whole embryo and radicle were taken as subsequent experimental materials.

Mean germination time (MGT) was calculated according to Ellis and Roberts [43]. Mean germination time (days) = Σ(nd)/Σn, where n was number of germinated seeds (2 mm radicle growth through seed coat) in day, d, of counting seed germination, and Σn was total germinated seeds.

### 4.6. Isolation and Purification of Mitochondria

Mitochondria were extracted with 80 embryos collected from seeds with radicles protruding 5 mm after imbibition for 66 h (aged seeds) and 42 h (non-aged seeds). All extraction procedures were carried out at 0~4 °C. The embryos were ground with a mortar and pestle, using a grinding medium. The grinding medium was composed of 50 mM phosphate buffer (pH 7.5), 0.3 M mannitol, 0.5% (*w*/*v*) bovine serum albumin (BSA), 0.5% (*w*/*v*) polyvinylpyrrolidone-40, 0.2 mM EDTA-2Na, and 20 mM cysteine. The homogenate was centrifuged at 2000× *g* for 10 min approximately twice. The supernatant was centrifuged at 12,000× *g* for 15 min. The precipitate was suspended in a wash medium buffer containing 0.3 M mannitol, 0.1% (*w*/*v*) BSA, and 10 mM N-[Tris (hydroxymethyl) metyl]-2-aminopropanesulfonic acid (TES) (pH 7.5), and centrifuged again at 12,000× *g* for 15 min. The final precipitate was washed once with a wash medium and suspended in a small volume of the medium (mitochondrial fraction). The crude mitochondria extract was used for enzyme activity determination.

For pure mitochondria, the suspension was loaded onto a Percoll step gradient consisting of 1:4:2 ratios, bottom to top, of 40% Percoll: 21% Percoll: 16% in a mannitol wash buffer. The mixture was centrifuged for 1 h at 40,000× *g*, and the mitochondria presented as an opaque band at the 21/40% and 16/21% interface. The mitochondrial band was collected and washed three times by centrifugation at 20,000× *g* for 15 min in a wash buffer containing 0.3 M mannitol, 0.1% (*w*/*v*) BSA, and 10 mM TES (pH 7.5), and the last time without BSA in the wash buffer.

### 4.7. Determination of H_2_O_2_ Content

The H_2_O_2_ content in the mitochondria of the embryo was carried out using a commercial chemical assay kit (Nanjing Jianchen Bioengineering Institute, Nanjing, China) according to the manufacturer’s instruction.

### 4.8. Enzyme Assays

Determination of the protein content in the mitochondria of the embryo was carried out using a commercial chemical assay kit (Suzhou Comin Biotechnology Institute, Suzhou, China) according to the manufacturer’s instruction. The assay was based on the Coomassie brilliant blue G-250 bound to protein to form a blue complex in the acidic solution, which shows a maximum absorption peak at 595 nm.

Catalase (CAT) (EC 1.11.1.6) activity in the mitochondria of the embryo was measured by the dynamic change in absorbance at 240 nm after 1 min, due to the decline of extinction of H_2_O_2_. A total of 20 μL of the supernatant was mixed with 800 μL phosphate buffer (25 mM, pH 7.0, mixed with 0.1 mM EDTA) and 200 μL 100 mM H_2_O_2_.

GR (EC 1.6.4.2) in the mitochondria of the embryo was carried out using a commercial chemical assay kit (Suzhou Comin Biotechnology Institute, Suzhou, China) according to the manufacturer’s instruction. One GR activity unit was defined as the decreasing rate of absorbance of 1 nmol NADPH per min at 340 nm.

DHAR (EC 1.8.5.1) in the mitochondria of the embryo was carried out using a commercial chemical assay kit (Suzhou Comin Biotechnology Institute, Suzhou, China) according to the manufacturer’s instruction. One DHAR activity unit was defined as the increase in absorbance of 1 nmol AsA per min at 265 nm.

MDHAR (EC1.6.5.4) in the mitochondria of the embryo was carried out using a commercial chemical assay kit (Suzhou Comin Biotechnology Institute, Suzhou, China) according to the manufacturer’s instruction. One MDHAR activity unit was defined as the oxidation of 1 nmol NADPH per min at 340 nm.

Complex IV in the mitochondria of the embryo was carried out using a commercial chemical assay kit (Suzhou Comin Biotechnology Institute, Suzhou, China) according to the manufacturer’s instruction. A complex IV activity unit was defined as the catalytic degradation of 1 nmol of reductive cytochrome c per min at 550 nm.

NAD-MDH in the mitochondria of the embryo was carried out using a commercial chemical assay kit (Suzhou Comin Biotechnology Institute, Suzhou, China) according to the manufacturer’s instruction. One NAD-MDH activity unit was defined as the consumption of 1 nmol NADH per min at 340 nm.

### 4.9. Protein Quantification and Digestion

Pure mitochondrial protein concentration was measured using the Bradford method. The appropriate amount of protein sample was mixed with 8 M urea solution to be quantified. According to the quantitative results above, the protein sample (500 µg each sample) was reduced by 10 mM DTT at 37 °C for 2.5 h and IAA was added to the final concentration of 10mM at room temperature for 40 °C min in darkness. All samples were transferred in the filter of a centrifuge tube (MW cutoff was 10 kDa). After reduction with DTT and alkylation with iodoacetamide, the proteins on the filter were washed three times using a lysis buffer and ABC solution (0.05 M NH4HCO3 in water), respectively. Then, the samples were digested by trypsin (1 μg trypsin for 100 μg protein) and incubated at 37 °C for 16 h. Subsequently, the peptide samples were used for LC-MS/MS analysis.

### 4.10. Mass Spectrometry Method and Data Analysis

#### 4.10.1. DDA Sample Acquisition

In order to generate the spectral library, peptides from each sample were mixed and acquired twice with a data dependent acquisition (DDA) mode using Q Exactive HF (Bremen, Germany, Thermo Fisher). The peptide mixture was separated using an EasyNano LC1000 system (San Jose, Thermo Fisher) with a home-made C18 column (3 µm, 75 µm × 15 cm) at a flow rate of 450 nL/min. A 120-min linear gradient was set as follows: 3% B(0.1% FA in H_2_O)/97% A(0.1% FA in H_2_O) to 6% B in 12 min; 6% B to 22% B in 75 min; 22% B to 35% B in 20 min; 35% B to 100% B in 6 min; and 7 min for 100% B. For the data acquisition, a top 20 scan mode with MS1 scan range *m*/*z* 400–1200 was used, and other parameters were set as below: MS1 and MS2 resolution was set to 120 K and 30 K; AGC for MS1 and MS2 was 3 × 10^6^ and 1 × 10^6^; isolation window was 2.0 Th; NCE was 27; and dynamic exclusion time was 20 s.

#### 4.10.2. Spectral Library Generation

DDA raw files were searched against a Uniprot protein database containing all plant proteins (downloaded on 2017.10.12, 2,304,711 entries) using Proteome Discoverer 2.1 (San Jose). The protein sequence was appended with the iRT fusion protein sequence (Biognosys, Schlieren, Switzerland). A search engine of SequestHT was used with the following searching parameters: enzyme of trypsin with maximum number of two missed cleavages; precursor and fragment ion mass tolerance was set to 10 ppm and 0.02 Da; variable modification was set to Oxidation of M, deamination of N, Q, Acetylation of Protein N terminus and fixed modification was set to carbamidomethylation of C; an algorithm of Percolator [44] was used to keep peptide FDR less than 1% and the q-value used for protein identification was 0.01. The search results of data-dependent acquisition using Proteome Discoverer 2.1 was transferred into a spectral library using Spectronaut 10 (Biognosys). Only a high confidence of peptide was used for the generation of the spectral library. Fragment ions within the mass range of *m*/*z* 300–1800 were kept and peptides less than three fragment ions were removed.

#### 4.10.3. DIA Sample Acquisition

Each sample, with the addition of the same amount of iRT, was analyzed using a data independent acquisition (DIA) method. This method consisted of one full MS1 scan, with resolution set at 60 K using AGC of 3 × 10^6^ and a maximum injection time of 20 ms. Sequential 29 isolation mass windows were set as follows: for *m*/*z* 400 to 800, the mass isolation window was set to 20 Th; for *m*/*z* 800–1000, the mass isolation window was set to 40 Th; and for *m*/*z* 1000–1200, the mass isolation window was set to 50 Th. Each DIA MS2 spectrum was acquired using a resolution of 30 K and AGC was set to 1 × 10^6^, maximum injection time was 45 ms and collision energy was set to NCE 30. All the LC conditions were exactly the same as the DDA sample acquisition listed above.

#### 4.10.4. DIA Data Analysis

DIA raw data were processed using Spectronaut 10. Default settings were used for protein identification and quantitation. Peak detection, dynamic iRT, correction factor 1, interference correction, and cross run normalization, were enabled. All peptides were filtered using a *Q* value ≤ 0.01. The average quantity of fragment ion areas from the top three peptides were used to compare protein abundance between samples. 

#### 4.10.5. Identification of Mitochondrial Proteins

Mitochondrial protein analysis was conducted when the quantitative proteins were screened for mitochondrion markers. At the same time, all proteins were submitted to DAVID (https://david.ncifcrf.gov/) and to GO annotation. Mitochondrial proteins were enriched in the mitochondria related pathways by analyzing the Cellular Component. In the study, proteins with a fold change of >1.2 or <0.8 (*p*-value ≤ 0.05) were regarded as differentially expressed proteins. The functions of these differential proteins were used as Basic Local Alignment Search Tools (BLAST) to find regions of local similarity between sequences in The *Arabidopsis* Information Resource (TAIR). Then, MapMan (MapManlnst-3.5.1 R2) was used to classify the functions of those mitochondrial proteins.

### 4.11. Statistical Analyses

The mean of three or four replicates was analyzed using analysis of variance (ANOVA), which was performed using SPSS 23.0 software. Duncan’s multiple range test was used to compare the treatment means of germination, physiological indicators and enzyme activities. Data was presented as means ± SD from three or four replications for each treatment. Different letters indicated significant differences among NO treatments (*p* < 0.05).

## Figures and Tables

**Table 1:** The germination percentage, mean germination time, and seedling length of aged oat seeds with different NO concentrations. Data are means ± standard deviation (SD) from four replications for each treatment. Different letters indicate significant differences among NO treatments (*p* < 0.05).

Treatment	Germination Percentage (%)	Mean Germination Time (d)	Root Length (cm)	Shoot Length (cm)
CK	99 ± 1.2 ^a^	1.9 ± 0.04 ^d^	6.2 ± 0.30 ^a^	4.7 ± 0.38 ^a^
A0	68 ± 5.9 ^b,c^	3.4 ± 0.14 ^b^	3.9 ± 0.50 ^c^	1.9 ± 0.23 ^c^
A1	78 ± 4.3 ^b^	3.0 ± 0.04 ^c^	5.1 ± 0.54 ^b^	2.4 ± 0.46 ^b^
A2	75 ± 8.3 ^b,c^	3.3 ± 0.20 ^b^	4.1 ± 0.40 ^c^	2.0 ± 0.27 ^b,c^
A3	70 ± 8.2 ^b,c^	3.4 ± 0.29 ^b^	2.0 ± 0.39 ^d^	0.7 ± 0.08 ^d^
A4	65 ± 6.2 ^c^	3.9 ± 0.19 ^a^	1.7 ± 0.05 ^d^	0.6 ± 0.08 ^d^

**Table 2:** Total of 52 differentially expressed proteins across three comparison groups in aged oat seeds in response to exogenous NO treatment.

Hit Number	Accession No.	Protein Name (Species)	Fold Change		
			A1/A0	A0/CK	A1/CK
TCA cycle					
I1LYN0	AT2G20420	Succinate-CoA ligase [ADP-forming] subunit beta, mitochondrial *Glycine max*	1.25	0.67	ns
K3ZV34	AT5G23250	Succinate-CoA ligase [ADP-forming] subunit alpha, mitochondrial *Setaria italica*	1.33	0.76	ns
Q10LR5	AT5G50950	Fumarate hydratase 1, mitochondrial, putative, expressed *Oryza sativa* subsp. *japonica*	1.25	ns	2.56
R4X771	AT5G50950	Fumarate hydratase, mitochondrial *Taphrina deformans*	ns	0.40	0.49
K3ZRI5	AT2G13560	Malic enzyme *Setaria italica*	ns	0.70	0.71
K3YRJ0	AT1G79440	Aldehyde dehydrogenase *Setaria italica*	ns	0.77	0.73
B4FRH5	AT2G20420	Succinate-CoA ligase [ADP-forming] subunit beta, mitochondrial *Zea mays*	ns	0.79	ns
W5C4B7	AT5G23250	Succinate-CoA ligase [ADP-forming] subunit alpha, mitochondrial *Triticum aestivum*	ns	0.67	ns
V4KMJ1	AT5G66760	Succinate dehydrogenase [ubiquinone] flavoprotein subunit, mitochondrial *Eutrema salsugineum*	ns	1.52	ns
M3AS20	AT5G40650	Succinate dehydrogenase [ubiquinone] iron-sulfur subunit, mitochondrial *Pseudocercosporafijiensis*	ns	0.74	ns
Mitochondrial electron transport					
A0A200QHI3	AT3G52300	ATP synthase subunit d, mitochondrial *Macleaya cordata*	0.80	ns	0.67
W5BEP1	AT3G52300	ATP synthase subunit d, mitochondrial *Triticum aestivum*	ns	2.20	2.37
A0A0K9R2N3	AT3G52300	ATP synthase subunit d, mitochondrial *Spinacia oleracea*	ns	1.24	ns
V4LNR7	AT3G52300	ATP synthase subunit d, mitochondrial *Eutrema salsugineum*	ns	ns	0.70
A0A1J3KAQ0	AT5G08530	NADH dehydrogenase [ubiquinone] flavoprotein 1, mitochondrial *Noccaea caerulescens*	ns	0.72	0.72
W5I0L9	AT3G03070	NADH dehydrogenase [ubiquinone] iron-sulfur protein 6, mitochondrial *Triticum aestivum*	ns	ns	0.68
Q9SKT7	AT5G13430	External alternative NAD(P)H-ubiquinone oxidoreductase B4, mitochondrial *Arabidopsis thaliana*	ns	3.93	4.23
K3Y9D3	AT5G13430	Cytochrome b-c1 complex subunit Rieske, mitochondrial *Setaria italica*	ns	ns	0.78
Protein synthesis and elongation					
A0A0D3H000	AT4G11120	Elongation factor Ts, mitochondrial *Oryza barthii*	1.35	0.65	ns
B6T7S2	AT5G47320	40S ribosomal protein S19 mitochondrial *Zea mays*	ns	0.59	0.58
F2DG12	AT2G45030	Elongation factor G, mitochondrial *Hordeum vulgare* subsp. *vulgare*	1.33	0.46	0.66
Q5JNL6	AT1G51980	Mitochondrial processing peptidase *Oryza sativa* subsp. *japonic*a	ns	ns	0.68
B6U5I0	AT2G29530	Mitochondrial import inner membrane translocase subunit Tim10 *Zea mays*	ns	0.72	0.62
Q6EN45	AT5G53140	Probable protein phosphatase 2C member 13, mitochondrial *Oryza sativa* subsp. *japonic*a	ns	0.42	0.62
A0A0D3GV84	AT5G26860	Lon protease homolog, mitochondrial *Oryza barthii*	ns	0.66	0.75
Q8L7B5	AT2G33210	Chaperonin CPN60-like 1, mitochondrial *Arabidopsis thaliana*	ns	ns	1.49
F2EDF6	AT4G11120	Elongation factor Ts, mitochondrial *Hordeum vulgare* subsp. *vulgare*	ns	0.74	ns
W5C618	AT5G26860	Lon protease homolog, mitochondrial *Triticum aestivum*	ns	0.76	ns
Signalling, G-protein					
F2CSX0	AT5G27540	Mitochondrial Rho GTPase *Hordeum vulgare* subsp. *vulgare*	ns	ns	0.75
A0A1Q3B5G5	AT5G27540	Mitochondrial Rho GTPase *Cephalotus follicularis*	ns	0.40	0.42
F2E3Y6	AT5G39900	Translation factor GUF1 homolog, mitochondrial *Hordeum vulgare* subsp. *vulgare*	ns	0.76	ns
RNA transcription					
Q7X745-2	AT5G39840	Isoform 2 of ATP-dependent RNA helicase SUV3L, mitochondrial *Oryza sativa* subsp. *japonic*a	1.33	0.62	ns
Q6K7E2	AT2G44020	Mitochondrial transcription termination factor-like *Oryza sativa* subsp. *japonic*a	ns	0.73	ns
Tetrapyrrole synthesis, protoporphyrin IX oxidase					
W5GSR7	AT1G48520	Glutamyl-tRNA(Gln) amidotransferase subunit B, chloroplastic/mitochondrial *Triticum aestivum*	ns	0.77	0.77
K3Y6C6	AT5G14220	Protoporphyrinogen oxidase *Setaria italica*	0.75	2.43	1.83
Stress, abiotic, heat					
G2X6B5	AT5G22060	Mitochondrial protein import protein MAS5 *Verticillium dahliae*	ns	1.56	1.66
A0A1J3J8H0	AT5G22060	Heat shock 70 kDa protein 10, mitochondrial (Fragment) *Noccaea caerulescens*	ns	0.23	0.28
Cell devision					
F2DZF0	AT3G57090	Mitochondrial fission 1 protein *Hordeum vulgare* subsp. *vulgare*	ns	1.30	1.25
W4ZR59	AT3G57090	Mitochondrial fission 1 protein *Triticum aestivum*	ns	0.73	0.68
Transport metobolism					
Q5NAJ0	AT1G14560	Graves disease mitochondrial solute carrier protein-like *Oryza sativa* subsp. *japonic*a	ns	1.30	1.25
Q10QM8	AT5G64970	Mitochondrial carrier protein, expressed *Oryza sativa* subsp. *japonic*a	ns	ns	0.68
Misc, oxidase-copper, flavone					
B4G146	AT5G06580	d-lactate dehydrogenase [cytochrome] mitochondrial *Zea mays*	0.69	1.58	ns
Amino acid metabolism					
B6SWZ4	AT4G34030	Methylcrotonoyl-CoA carboxylase beta chain mitochondrial *Zea mays*	ns	ns	1.21
Co-factor and vitamine metabolism, lipoic acid					
U5H066	AT5G08415	Lipoyl synthase, mitochondrial *Microbotryum lychnidis-dioicae*	ns	0.73	0.72
Redox, Glutaredoxins					
Q0JQ97	AT3G15660	Monothiol glutaredoxin-S1, mitochondrial *Oryza sativa* subsp. *japonic*a	ns	0.78	ns
Nucleotide metabolism					
K3ZE81	AT5G23300	Dihydroorotate dehydrogenase (quinone), mitochondrial *Setaria italica*	ns	ns	0.75
C1-metabolism formate dehydrogenase					
A0A0D3GGT7	AT5G14780	Formate dehydrogenase, mitochondrial *Oryza barthii*	ns	0.72	0.67
Major CHO metabolism, Degradation, sucrose, Invertases, nautral					
Q10MC0	AT1G56560	Neutral/alkaline invertase 1, mitochondrial *Oryza sativa* subsp. *japonic*a	ns	0.76	ns
Not assigned					
W5BQ98	AT4G22310	Mitochondrial pyruvate carrier *Triticum aestivum*	1.31	ns	ns
Q6ZGV8	AT3G52140	Clustered mitochondria protein homolog *Oryza sativa* subsp. *japonic*a	ns	1.68	1.91
Q0JDA2	AT1G47420	Succinate dehydrogenase subunit 5, mitochondrial *Oryza sativa* subsp. *japonic*a	ns	1.32	1.23
B6SPH3	AT5G08040	Mitochondrial import receptor subunit TOM5-like protein *Zea mays*	1.45	0.33	0.45

Different regulated proteins regarded as the abundance was equal to or more than 1.2-fold or less than 0.8-fold (*p* ≤ 0.05). The “ns” indicates no significant difference.

## Abbreviations

CAT	Catalase
DHAR	Dehydroascorbate reductase
ETC	Electron transport chain
GR	Glutathione reductase
H_2_O_2_	Hydrogen peroxide
MGT	Mean germination time
MDHAR	Monodehydroascorbate reductase
NAD-MDH	NAD-malate dehydrogenase
TCA	Tricarboxylic acid
